# Accentuating the Positive: Do Trials Give Unrealistic Expectations of Long-Term Survival?

**DOI:** 10.1093/jncics/pkz018

**Published:** 2019-04-15

**Authors:** Belinda E Kiely, Martin R Stockler

**Affiliations:** 1 NHMRC Clinical Trials Centre, The University of Sydney, Camperdown, NSW, Australia; 2 Concord Cancer Centre, Concord Repatriation General Hospital, Concord, NSW, Australia

When diagnosed with an incurable cancer, many patients hope for a miracle and cling to stories of long-term survivors, hoping that they too will be one who defies the odds and beats their cancer. As oncologists, we too are excited by new treatments that can improve survival, and eagerly await the opportunity of using them in our own patients. Trials in stage IV non-small-cell lung cancer (NSCLC) during the last decade have demonstrated that new treatments can substantially improve overall survival beyond the median of 9–12 months observed with first-line, platinum-based chemotherapy during the previous two decades (1). Median overall survival is measured in years for patients treated with targeted therapies for lung cancers with driver mutations and for some lung cancer patients treated with immunotherapy. For example, the median survival for patients with 50% or higher PDL1 expression receiving first-line single agent pembrolizumab was 30 months (2). But does this mean we can expect similar survival outcomes for the patients we see and treat in routine clinical practice? The answer to this question depends on knowing how similar our patients in routine practice are to those enrolled in pivotal clinical trials.

The strict eligibility criteria of pivotal randomized clinical trials can limit the generalizability of their results (3). Trial participants have fewer adverse events and lower mortality rates than nonparticipants (4), mainly due to differences in their baseline characteristics and comorbidities rather than differences in treatment. Patients in routine clinical practice who meet the eligibility criteria for a clinical trial, and are treated like those in the trial, might reasonably expect similar survival outcomes to trial participants. However, due to the presence of comorbidities, poor performance status, brain metastases, concomitant medications, and other factors, many patients in routine practice would not be eligible for these trials and cannot reasonably expect similar outcomes.

The accompanying article by Davis et al. (5) reports on the overrepresentation of long-term survivors in clinical trials compared with an unselected population and warns us to consider this when extrapolating the survival outcomes from trials to all patients in routine practice. Using the Surveillance Epidemiology and End Results (SEER) database to obtain a population-based cohort of 44 387 patients diagnosed with stage IV NSCLC between 1991 and 2007, the authors characterized the long-term survivors, whom they defined either as the 10% surviving the longest or those surviving at least 5 years. The median survival from diagnosis for the whole SEER cohort was only 4 months, but 10% survived 21 months or longer and 2% survived at least 5 years. Although survival improved over the 16-year study period, most of this improvement was seen for the 10% of long-term survivors (in whom median survival increased from 30 to 36 months), with a much smaller absolute improvement among the remaining 90% (in whom median survival increased from 3 to 4 months).

To compare these findings to those in a clinical trial, the authors evaluate the survival of 165 patients with stage IV NSCLC enrolled in the CUSTOM clinical trial (6) investigating the effects of targeted agents against speciﬁc molecular aberrations. In the CUSTOM trial, the median survival from diagnosis was 27 months among all participants, with 54% living 21 months or longer (compared with 10% in the SEER cohort) and 4% surviving 5 years or longer (compared with 2% in the SEER cohort).

An approximately 7-fold difference in median survival between the CUSTOM trial and the SEER cohort emphasizes the need for caution extrapolating survival outcomes from clinical trials to routine practice. However, the small and more similar proportions of patients surviving 5 years or longer reminds us that a few patients will do exceptionally well, regardless of their treatment.

Although Davis et al. conclude that long-term survivors are overrepresented in clinical trials, other factors may contribute to the longer survival seen in the trial population. There was limited information about baseline characteristics in the SEER cohort, so it is impossible to determine the importance of differences in risk factors, comorbidities, performance status, and access to health care. The trial patients were diagnosed more recently and are likely to have had access to newer and perhaps more effective treatments that were not available for the SEER cohort. We also do not have any details on the treatments received, if any, by the SEER population, although it is likely to be predominantly chemotherapy or best supportive care, both associated with shorter survival than the targeted therapies received by many of the trial participants.

We cannot predict any individual’s survival time with certainty, but we can provide ranges for likely survival times based on the outcomes of individuals with similar characteristics and treatments. For patients who meet clinical trial eligibility criteria, the reported median survival from a clinical trial is a good starting point. However, for the many patients who do not meet clinical trial eligibility criteria, survival estimates based on pivotal trials will need to be adjusted down. Unfortunately, there are limited data to guide clinicians trying to make these adjustments. Registries collecting outcome data for patients receiving treatments outside of clinical trials would help address this problem, but few such registries exist.

Our work in metastatic breast cancer (MBC) comparing survival outcomes of patients starting first-line chemotherapy in routine clinical practice (7,8) and in clinical trials (9,10) may help. For a mixed population with HER2-negative, HER2-unknown, and HER2-positive MBC starting first-line chemotherapy, the median survival was 20 months in routine practice (7) and 22 months in clinical trials (9). For patients with HER2-positive MBC starting first-line chemotherapy and trastuzumab, the median survival was 30 months in routine practice (8) and 33 months in clinical trials (10). Although the median survival was shorter for the routine practice patients compared with the trial participants, the difference was much smaller than that reported by Davis et al. (4 vs 27 months). In MBC, we also found that the differences in survival were largest for the patients surviving the shortest times, with smaller differences in longer survivors, probably reflecting the exclusion from clinical trials of patients expected to have short survival times (eg, <3 months). MBC is a very different disease than NSCLC, but our results imply that the adjustment of survival times in clinical trials required to represent patients in routine care receiving the same treatment need only be small. Of note, we only reported the survival of MBC patients treated with chemotherapy, which was 54% of the women in our mixed MBC cohort (7). The survival for “all comers” in that MBC population, including those not treated with chemotherapy, would be much shorter.

Patients we surveyed overwhelmingly preferred to receive information on their expected survival time formatted as ranges for three scenarios (worst-case, best-case, and typical) rather than as a single point estimate of median survival (11). We have previously shown how these three scenarios for survival can be calculated from an oncologist’s estimate of a patient’s expected survival time based on the median in a group of similar patients (12,13). Providing patients with ranges for three scenarios for survival helps them prepare for the possible worst-case, hope for a realistic and equally possible best-case, and plan for the more likely typical scenario. Quantifying a best-case scenario as the survival of the 10% of similar patients surviving the longest provides a more realistic and probable hope than describing a few, exceptionally long survivors.

In our frequent conversations about prognosis with patients, we often struggle with the tension between maintaining hope and being honest enough to help people plan for the future. We want our patients to be exceptional responders, perhaps as much as our patients hope for this. Telling patients about the survival outcomes reported in clinical trials is an obvious and appropriate starting point when discussing life expectancy. However, such survival times are probably unrealistic for the many patients seen in routine practice who would not meet the eligibility criteria for that trial. More research is needed to help oncologists better estimate survival for patients who would not be eligible to participate in the trial that established their proposed treatment. Until then, adjusting the median survival from the clinical trial down by a few months and converting this adjusted estimate into ranges for worst-case, best-case, and typical scenarios for survival enables communication of prognostic information that allows for realistic hope and realistic plans.

## Notes

Affiliation of authors: NHMRC Clinical Trials Centre, The University of Sydney, Camperdown, NSW, Australia (BEK, MRS); Concord Cancer Centre, Concord Repatriation General Hospital, Concord, NSW, Australia (BEK, MRS).

BEK and MRS have no conflicts of interest related to this publication.
